# Loss of empathy in stroke

**DOI:** 10.3389/fpsyg.2024.1451431

**Published:** 2024-11-25

**Authors:** Wai Kwong Tang, Edward Hui, Thomas Wai Hong Leung

**Affiliations:** ^1^Department of Psychiatry, The Chinese University of Hong Kong, Shatin, Hong Kong SAR, China; ^2^Department of Imaging and Interventional Radiology, The Chinese University of Hong Kong, Shatin, Hong Kong SAR, China; ^3^Department of Medicine and Therapeutics, The Chinese University of Hong Kong, Shatin, Hong Kong SAR, China

**Keywords:** stroke, empathy, executive function, MRI, prefrontal cortex, anterior insula, amygdala, thalamus

## Abstract

**Background:**

Loss of empathy (LoE) is common among stroke survivors, yet often undiagnosed and thus untreated. LoE is related to the loss of a caring marital relationship, higher care burden and poorer quality of life in carers. The present study will evaluate the clinical and MRI correlates of LoE in a cohort of stroke survivors. The secondary objective is to describe the 12-month course of LoE.

**Methods:**

The current study is a prospective cohort study. We will recruit 246 subjects. Subjects and carers will receive a detailed assessment at a research clinic at 3, 9, and 15 months after stroke onset (T1/T2/T3). The Chinese version of the Interpersonal Reactivity Index (IRI), a 28-item personality assessment tool, will be completed by a carer for each subject. LoE is defined as an IRI total score of 39 or less. Patients will be examined by MRI including diffusion weighted imaging (DWI) within 1 week after the onset of stroke. A stepwise logistic regression will be performed to assess the importance of lesions in the regions of interest. To examine the predictors of LoE remission, the demographic, clinical and MRI variables of remitters and non-remitters at T2/T3 will be examined by logistic regression.

**Discussion:**

This project will be the first longitudinal study on LoE in stroke survivors. The results will shed light on the association between prefrontal cortex and subcortical lesions and LoE risk, symptom severity and outcome. The findings will provide data to advance our understanding of the pathogenesis and clinical course of LoE in stroke as well as other neurological conditions. They are thus likely to be applicable to the large population of neurological patients at risk of LoE and should also stimulate further research in this field.

## Background

Loss of empathy (LoE) is defined as a lack of ability to recognize, share and make inferences about another person’s emotional state (Hillis, 2013). Empathy is a social cognitive function (Henry et al., 2015) that is fundamental for the success of human relationships and communication (Jiang et al., 2014). Empathy consists of emotional and cognitive aspects. Emotional empathy refers to the ability to recognize and share the feelings of another person, whereas cognitive empathy is the ability to make inferences about what another person is thinking or feeling (Shamay-Tsoory, 2010). Family members of patients with LoE often describe them as selfish, self-centered and with an obvious lack of concern for the feelings and distress of others (Hsieh et al., 2013).

LoE is common in cerebral diseases such as brain tumor (Herbet et al., 2015), frontotemporal dementia (Oliver et al., 2015), traumatic brain injury (Bivona et al., 2014) and stroke (Oishi et al., 2014). For instance, 56% of patients with cerebral lesions scored more than 2 standard deviations below the mean on empathy tests (Grattan and Eslinger, 1989). In patients with severe head injury, 55–64% had low empathy (Williams, 2009).

LoE contributes to social behavioral difficulties and interpersonal and communication problems in patients with head injury (Saxton et al., 2013). It is one of the most frequent and important residual symptoms in stroke survivors (Hillis and Tippett, 2014). Among carers, LoE is also related to the loss of a caring marital relationship (Hsieh et al., 2013), higher care burden (Hsieh et al., 2013) and poorer quality of life (Wells et al., 2005). Therefore, LoE could be a focus of stroke rehabilitation (Williams, 2009).

LoE appears to be common among stroke survivors; one study reported that 15 out of 30 survivors were affected (Oishi et al., 2014). However, the prevalence of LoE in local stroke survivors is unknown because there is a lack of large-scale studies. The clinical correlates of LoE in stroke include older age, reduced cognitive flexibility (Eslinger et al., 2002) and emotional processing and theory of mind (ToM) capabilities, but not sex or volume of infarcts (Eslinger et al., 2002; Leigh et al., 2013). Correlates of LoE in other neurological disorders and healthy volunteers include female sex (Weddell and Wood, 2016), younger age (Tran et al., 2013), severity of disease (Hsieh et al., 2013), depression (O’Keeffe et al., 2007), and anosmia (Weddell and Wood, 2016). The course of LoE in stroke is uncertain. Our previous research data show that the 12-month non-remission rate of post-stroke depression (PSD), another neuropsychiatric condition, is 66%; the clinical correlates of persistent PSD are baseline severity of depression, severity of stroke and cognitive functioning (Tang et al., 2014).

Studies on healthy individuals and lesion studies have suggested that the following areas are important for empathy: ventromedial prefrontal cortex (VMPC), orbitofrontal cortex (OFC), inferior frontal cortex (IFC), anterior cingulate cortex (ACC), anterior insula, anterior temporal cortex (ATC), amygdala and thalamus (Hillis and Tippett, 2014). Specifically, the right IFC and OFC are critical for emotional empathy, the right VMPC is critical for cognitive empathy and the right anterior insula, ACC, ATC and amygdala are critical for both (Hillis and Tippett, 2014). The relationship between LoE and dysfunctions in the above regions are discussed in the following paragraphs.

The prefrontal cortex is involved in cognitive empathy (Hooker et al., 2008). Most lesion studies report that right or left prefrontal lesions, especially the ventromedial area, are associated with impairments in cognitive empathy (Hillis and Tippett, 2014). Functional imaging and lesion studies provide evidence for the role of the IFC and OFC, especially on the right, in emotional empathy (Jabbi et al., 2007; Shamay-Tsoory et al., 2009; Spikman et al., 2012). Functional imaging studies indicate that the perception of another person’s feelings engages the anterior insula and ACC (Bernhardt and Singer, 2012). Case reports of patients with anterior insular and cingulate lesions have shown impairments in empathy (Baird et al., 2006; Gu et al., 2012). LoE in patients with traumatic brain injury is related to lesion volume in the insula (Driscoll et al., 2012). Anterior insular and right ACC infarcts are related to impaired cognitive empathy in stroke (Leigh et al., 2013).

Voxel-based morphometry studies have indicated that the right ATC is likely to play a critical role in both emotional and cognitive empathy (Hillis and Tippett, 2014). Functional imaging studies show that empathy is associated with activation of the right amydala (Bzdok et al., 2012) and perception of others’ emotions with activation of the right thalamus (Gu et al., 2012). Leisons in the right or bilateral amydala consistently interfere with performance on empathy tasks (Hurlemann et al., 2010; Leigh et al., 2013). Our team has reported that thalamus lesions are linked to post-stroke emotional lability (Tang et al., 2009).

Very few structural brain imaging studies have been published on LoE in stroke (Grattan et al., 1994; Yeh and Tsai, 2014; Wilkos et al., 2015; Leigh et al., 2013; Oishi et al., 2014). Case reports have linked LoE in stroke to insular infarct (Couto et al., 2013). Small case control studies have found associations between LoE in stroke and leisons in the right hemisphere (Yeh and Tsai, 2014), OFC and MFPC (Shamay-Tsoory et al., 2004), ATC (Leigh et al., 2013), anterior insula (Leigh et al., 2013), and thalamus (Wilkos et al., 2015). The limitations of these studies include small sample sizes (Wilkos et al., 2015), very selected samples (Oishi et al., 2014), a lack of detailed examination of infarct locations (Yeh and Tsai, 2014) and no follow-up assessment (Wilkos et al., 2015).

There is no published longitudinal data on any detailed or systematic examination of the pattern, clinical course and MRI correlates of LoE in stroke populations. The objectives of the proposed study will be to evaluate the clinical, neuropsychological and MRI correlates and the 12-month course of LoE in a cohort of stroke survivors. The regions of interest (ROIs) are VMPC, OFC, IFC, ACC, ATL, anterior insula, amygdala and thalamus.

### Hypotheses

The first hypothesis is that subjects with LoE will have more infarcts in the ROIs, but not in the control region (occipital lobe), than subjects without LoE (Hillis and Tippett, 2014; Hooker et al., 2008; Jabbi et al., 2007; Shamay-Tsoory et al., 2009; Spikman et al., 2012; Bernhardt and Singer, 2012; Baird et al., 2006; Gu et al., 2012; Driscoll et al., 2012; Bzdok et al., 2012; Hurlemann et al., 2010; Tang et al., 2009; Grattan et al., 1994; Yeh and Tsai, 2014; Wilkos et al., 2015). The second hypothesis is that there will be a significant positive correlation between the number of infarcts in the ROIs and the severity of LoE (Tang et al., 2017). The third hypothesis is that subjects with LoE will have poorer emotional processing, theory of mind (ToM) and executive functioning (Yan et al., 2020; Yeh and Tsai, 2014). A positive correlation is predicted between the IRI total and subscale scores and measures of the above neuropsychological functions in the LoE group. The fourth hypothesis is that 66% (Tang et al., 2014) of subjects with LoE at baseline will continue to have LoE 12 months after the first assessment. The fifth hypothesis is that the baseline severity of LoE, severity of stroke and level of cognitive functioning will predict the persistence of LoE (Tang et al., 2014). The fourth hypothesis is that there is a correlation between the structural connectivity between the regions of interests and risk/severity/persistence of LoE.

## Methods

### Sample size estimation

Two hundred and forty-six patients will be recruited. As there are no published data on the location of infarcts in the risk and course of LoE in stroke, we calculated the sample size using the figures reported for another neuropsychiatric disorder, namely PSD. In a report on PSD, 25.4 and 25.4% of patients with PSD had frontal and temporal infarcts, respectively, compared with only 8.6 and 8.0% of patients without PSD (Metoki et al., 2016). Using these figures as the study estimate, a sample size of 246 will have 80 and 84% power in identifying frontal and temporal infarcts, respectively, as a predictor of LoE in stroke, using a chi square test with one degree of freedom (Cohen, 1988).

Power analysis was conducted using the Power Analysis and Sample Size Software (2024; NCSS, LLC, Kaysville, Utah, United States, ncss.com/software/pass). Using 50% (Oishi et al., 2014) as an estimate of the frequency of LoE, 123 (246 × 50%) cases will be identified. A sample size of 123 LoE subjects will provide 80% power (Zar, 1984) in detecting any correlate of the IRI score with a correlation of 0.13, a figure considerably lower than the reported associations between the severity of LoE and age (−0.20) (Gleichgerrcht et al., 2015), depressive symptoms (0.33) (Jiang et al., 2014) and executive functions (0.47–0.67) (Narme et al., 2013). Assuming a drop-out rate of 20%, 98 (123 × 80%) patients with LoE will attend the follow-ups. Based on a remission rate of 35–50% in PSD (Berg et al., 2003; Bour et al., 2010; Tang et al., 2014), this sample will provide 99% power in identifying any predictor with an odds ratio of 5.3, a figure reported in our previous work on cerebromicrobleeds and PSD outcomes (Tang et al., 2014), assuming that the R2 of the other variables = 0.21 (Tang et al., 2014) in the multivariate logistic regression (Cohen, 1988; Ferguson, 1959).

### Recruitment of subjects

**Figure 1:**
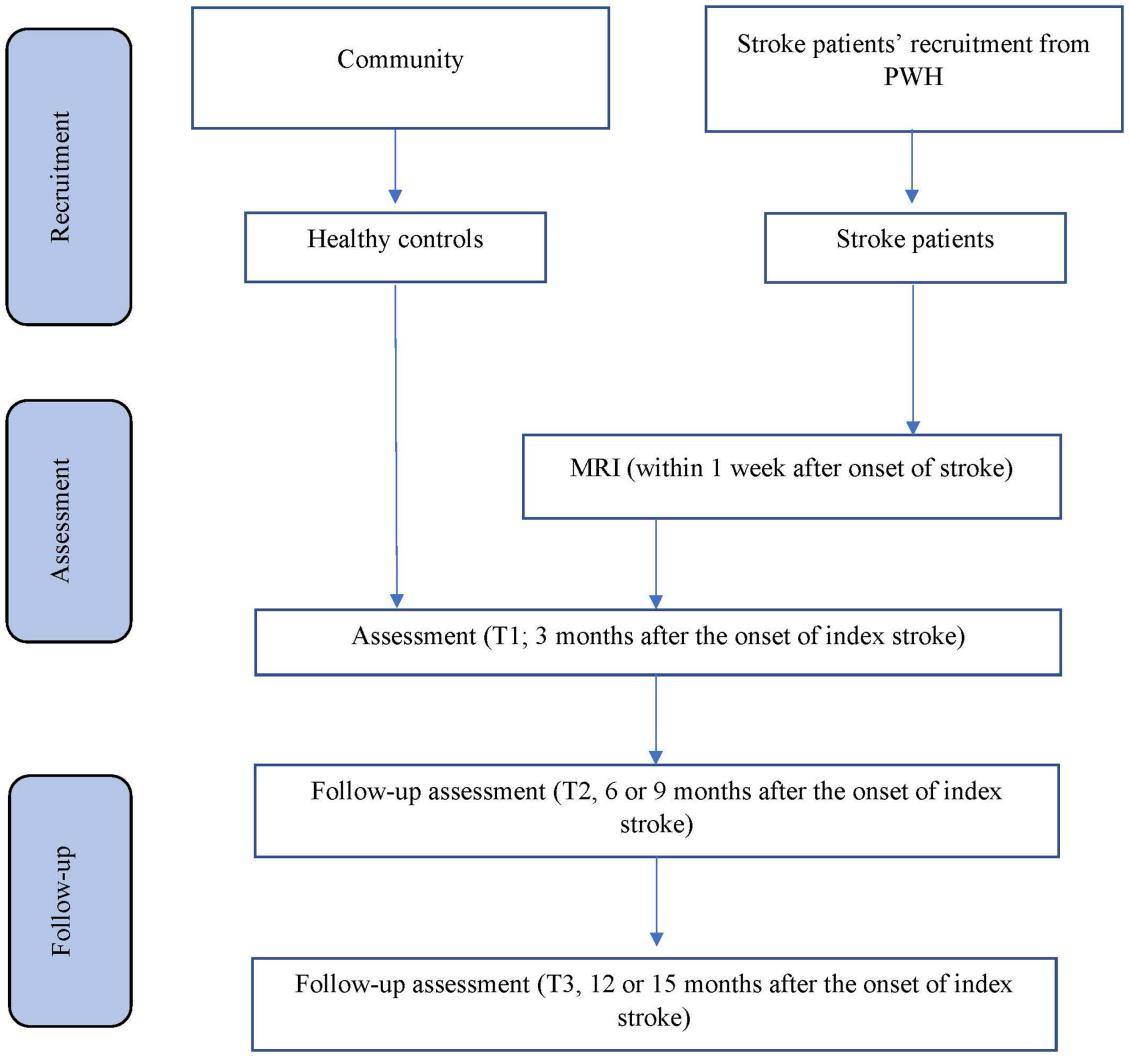
Details of recruitment.

The planned study is a prospective nested case–control study of stroke survivors. Details of recruitment are shown in Figure 1. Patients will be recruited from the Acute Stroke Unit (ASU) of the Prince of Wales Hospital in Hong Kong. The ASU treats approximately 93% of all acute stroke patients admitted to the hospital, with the majority of the remaining 7% sent to the neurosurgery unit. A research assistant will visit the ASU daily to identify all eligible patients and obtain their written consent. All acute stroke patients (*n* = 550) consecutively admitted to the ASU over a 12-month period will be invited to participate. A research assistant will visit the ASU daily to identify eligible patients and to obtain written consent. Approximately 80% of the 550 patients (440) will have ischemic stroke. MRI examination will be contraindicated in 10% of patients, leaving 396 potential subjects (440 × 90%). According to our previous findings (Tang et al., 2005), the mortality rate at 3 months post-stroke is around 12%, leaving 348 [396 × (100% − 12%)] potential subjects. Of these, 25% will not meet the inclusion criteria (Tang et al., 2005). Hence, the number of possible subjects will be around 261 [348 × (100% − 25%)] (Tang et al., 2005). Assuming a dropout rate of 20%, 209 [261 × (100 − 20%)] patients will complete the 12-month follow-up assessment. A healthy control group (*n* = 246) matched by age, sex and years of education will be recruited from the community. There is no overlap between the patient samples that will be included in the current manuscript and the other manuscript (Feng et al., 2024).

### Eligibility criteria

#### Inclusion and exclusion criteria

The inclusion criteria are (1) Age ≥ 18 years with no upper age limit, as the ASU only treats adult patients; (2) either male or female, in order to enhance generalizability of findings; (3) Chinese ethnicity, to increase homogeneity of the sample; (4) right handed, to facilitate interpretation of laterality of infarction locations; (5) well-documented acute first ischemic stroke affecting either the right or left hemisphere (Yeh and Tsai, 2014; Hillis and Tippett, 2014; Wilkos et al., 2015) occurring within a maximum of 7 days prior to admission, to ensure patients had an acute stroke; and (6) the ability and willingness to give informed consent, to meet research ethics standard.

The exclusion criteria are (1) Previous history of epilepsy, head injury, hydrocephalus, intracranial tumor, Parkinson’s disease, dementia or other neurological disease(s) other than stroke, to avoid other neurological causes of LoE; (2) history or current diagnosis of depression, bipolar disorder, schizophrenia, alcohol/substance abuse/dependence, autism and related disorders, to avoid other psychiatric causes of LoE; (3) dementia, defined as a Mini-Mental State Examination score below 20 or severe neuropsychological dysfunction that precludes detailed neuropsychological assessment, including aphasia, neglect, visual agnosia and dyslexia, as documented in the medical note (Yeh and Tsai, 2014); (4) contraindications for MRI examination and hence unavailability of MRI data for analysis, such as pacemaker *in situ*, physical frailty or severe claustrophobia; (5) without a caregiver to provide information on LoE assessment; and (6) recurrence of stroke prior to the 3-month assessment, as further stroke will complicate the lesion analysis. For the control group, the exclusion criteria include history of stroke.

### Data collection

Details of the data collection schedule are shown in Table 1. Written consent from patients or relatives (by proxy) will be obtained. The number of exclusions and reasons for them will be recorded. The following demographic, psychosocial and medical data of all participants will be collected: age, sex, education and date of onset of stroke. The subjects’ clinical data and information on neurological impairment, including dysarthria, measured by the National Institute of Health Stroke Scale (NIHSS) (Brott et al., 1989), will be extracted from the Stroke Registry, which is maintained by a full-time, well-trained research nurse.

**Table 1:** Data collection schedule.

Study period	–	T1	T2	T3
Visit	1	1	2	3
Months after index stroke/recruitment (for stroke subjects/healthy controls)	–	3	9	15
Demographics and clinical data	X	X		
Recognition of facial expression and ToM task	X	X		
The Color Trials Test; The Verbal Fluency Test; The Stroop Test	X	X		
Digit Span Test, HKLLT	X	X		
IRI, BI, BDI, MoCA	X	X	X	X

### Assessment of LoE

Three months after the onset of the index stroke (T1), the patients and their carers will receive the following assessments in a single session at a research clinic. The timing of the assessment is consistent with other studies of neuropsychiatric disorders in stroke (Tang et al., 2009; Tang et al., 2012). The evaluation will be conducted in a fixed order of the tests/tasks.

A psychiatrist blind to the subjects’ radiological data will conduct a clinical interview at a research clinic. LoE will be assessed with the Chinese version of the Interpersonal Reactivity Index (IRI). The IRI is based on an interview with a caregiver. The 28 items assess both cognitive and emotional empathy. Each item is answered on a 5-point Likert type scale ranging from “not true of me at all” to “frequently true of me.” The items are divided into four subscales: the perspective taking and fantasy subscales measure cognitive empathy, and the empathy concern and emotional distress subscales measure emotional empathy. The total score, derived by summing all four subscales, is used to define LoE. The mean (standard deviation) IRI total score in adult healthy controls is 62.0 (11.4) (Lam et al., 2014). LoE is defined as an IRI total score of 39 or less [2 standard deviations below the mean: 62 – (11.4 × 2) = 39.2] (Grattan and Eslinger, 1989; Leigh et al., 2013). The IRI has adequate internal reliability with alpha levels ranging from 0.65 to 0.70 and satisfactory test–retest reliability ranging from 0.68 to 0.83 (Siu and Shek, 2005). The IRI is a commonly used tool for the assessment of LoE in stroke (Shamay-Tsoory et al., 2009; Leigh et al., 2013; Yeh and Tsai, 2014; Ozzoude et al., 2021). The Dysexecutive Questionnaire (Wilson et al., 1996) contains no item to measure LoE. Other scales for measuring LoE, such as the Balanced Emotional Empathy Scale (Mehrabian and Epstein, 1972), have not been translated into and validated in Chinese. In addition, Empathy Quotient (EQ) (Baron-Cohen and Wheelwright, 2004), a self-report questionnaire that measures the cognitive, affective, and behavioral aspects of empathy. It contains 40 empathy items and 20 filler/control items. On each empathy item a person can score 2, 1, or 0, so the EQ has a maximum score of 80 and a minimum of zero.

A trained RA, blind to the subjects’ radiological data, will administer the following neuropsychological tests (Table 1).

### Assessment of emotional processing and ToM deficits

Two tasks will be administered to determine whether LoE is related to processing of emotional stimuli and ToM deficits.

1. Recognition of facial expression

The computer-based Facial Emotion Recognition paradigm (Yip et al., 2003; Chan et al., 2008) uses a set of male and female Asian faces adopted from the Japanese and Caucasian Facial Expressions of Emotion photoset (Ekman and Matsumoto, 1993). The set of photographs chosen for use in this paradigm has been found to have good agreement (male faces 81.4%; female faces 78.9%) in the local Chinese population (Yip et al., 2003). The 12 photographs are presented sequentially on a computer screen (Chan et al., 2008). Each photograph conveys one of the six basic emotions (e.g., happy, sad, anger, surprise, disgust, and fear). The dimensions of photographs are 6 inches × 8 inches. These photographs are presented individually for 10 s on a personal portable computer. These photographs are programmed in a computer using the E-Prime (Psychology Software Tools Inc., Pittsburgh, United States) software package. Subjects are required to indicate which emotions the faces in the photographs convey. The paradigm takes 10 min to complete. The number of correct answers and reaction times are recorded.

2. ToM task (Faux Pas Test) (Stone et al., 1998)

We will use 10 faux pas stories. Each contains a social faux pas that involves two or three characters, whose actions are described in at least two separate statements. Each story is read to the subject. Afterwards, the subject is asked the following five questions to check whether they:

- detected a faux pas;
- understood the faux pas;
- Understood the mental state of the faux pas’ recipient;
- understood the mental state of the person delivering the faux pas; and
- understood the details but without inferring the mental states of any of the characters in the story.

Participants who respond “no” to the first question skip questions 2–4 and are immediately presented with question 5. In this case, questions 2–4 are assigned zero points. We will record the responses of all subjects verbatim. We will assign one point for each correct response then produce scores for individual questions (sum of the respective scores obtained for the 10 stories) and a faux pas total score (sum of the scores for questions 1–4 for all 10 stories). The 3-month test–retest reliability of the Chinese version of the faux pas test is 0.83, and the interrater reliability is 0.76 (Zhu et al., 2007). It has been applied in stroke research (Kemp et al., 2013).

### Assessment of cognitive flexibility and executive functioning

To examine whether specific cognitive processes contribute to LoE, the following tests will be administered.

1. The Color Trails Test (Maj et al., 1993; D’Elia et al., 1996) is a timed assessment of visuomotor speed and executive functioning. It has been widely used in stroke research (Sakai et al., 2024).
2. The Verbal Fluency Test (Mok et al., 2004) requires subjects to generate as many animal, fruit and vegetable names as possible in 1 min. It measures speed and activation as well as executive processes including clustering, set-shifting and retrieval. It has been widely used in stroke research (Chen et al., 2013; Godefroy et al., 2024).
3. The Stroop Test (Stroop, 1992) is used to measure executive functioning. Subjects are presented with the names of colors written in different colored inks, and must name the color word not the color it is written in. The time to complete the task and the number of errors will be recorded. This test examines mental flexibility and the capacity to inhibit learned responses in favor of performing novel behavior. It has been widely used in stroke research (Chan, 2023; Heiberg et al., 2023).

### Assessment of basic cognitive functioning

1. The Hong Kong version of the Montreal Cognitive Assessment (MoCA) (Yeung et al., 2014) measures global cognitive functions. It has been validated and applied in local stroke patients (Wong et al., 2015; Ismail et al., 2022).
2. The Digit Span Test (Wechsler, 1997) assesses attention and working memory. The digit backward subscore will be used as the index of working memory, with scores ranging from 0 to 14 with higher scores reflecting superior performance. This test has been widely used in stroke research (Tamez et al., 2011).
3. The Hong Kong List Learning Test is used to assess memory (Chan and Kwok, 1999). The HKLLT is based on the California Verbal Learning Test. It presents 16 words in 3 learning trials, followed by a 10 min delayed recall and 30 min delayed recall and recognition test. It has been validated in both normal and clinical samples (Chan and Kwok, 1999). HKLLT has been applied in stroke research (Wong et al., 2020).

### Other assessments

A trained RA, blind to the subjects’ radiological data, will measure the level of physical functioning, apathy and depressive symptoms using the Barthel Index (BI) (Mahoney and Barthel, 1965), Apathy Evaluation Scale (AES) (Wang et al., 2008) and the Beck Depression Inventory (BDI) (Shek, 1990) and Geriatric Depression Scale (GDS) (Yesavage, 1988) respectively.

Follow-up assessments of LoE will be conducted on all stroke patients at 9 months (T2) and 15 months (T3) post-stroke, or 6 and 12 months after the first assessment. The assessments (IRI, BI, MoCA, AES, GDS, and BDI) will be repeated during the follow-up assessment.

Quality control measures will be implemented, including review of training logs to ensure that sufficient and relevant training has been completed and documented; conducting systematic comparison of the electronic (or paper, if applicable) clinical data to the medical records; reviewing the contents of the Essential Documents Binder and documenting the results of the review.

### Magnetic resonance imaging examination and analysis

Patients will be examined by MRI within 1 week after the onset of stroke. All scans will be performed using a 3 T scanner (Philips Achieva 3.0 T, X Series, Quasar Dual MRI System) with standardized sequences, including diffusion weighted imaging (DWI), 3D T1-weighted, T2-weighted, fluid attenuated inversion recovery (FLAIR) and susceptibility-weighted imaging (SWI). An experienced neuroradiologist blind to the subjects’ LoE status will assess the MRI images. Acute infarct will be defined as a hyperintensive lesion on DWI with corresponding hypointensity on the ADC map. White matter hyperintensities (WMH) will be defined as hyperintensities ≥5 mm that are ill defined on FLAIR images, but are isointense with normal brain parenchyma on T1 weighted images. Lesions equivalent to the signal characteristics of cerebrospinal fluid on T1-weighted images and measuring more than 3 mm in diameter, and also wedge-shaped cortico-subcortical lesions, will be regarded as old/lacunar infarcts. Microbleeds will be defined as dot-like hypointensities on SWI. The total number of microbleeds will be determined. All raw data will be transferred to the PALS system (Carestream Solutions). Diffusion MRI will be performed using the diffusion-weighted spin-echo echo-planar imaging sequence (b-values of 1,500 and 3,000 s/mm2 along 92 diffusion-encoding directions, multiband factor = 4, repetition time/echo time = 3,230/89 ms, field of view = 210 mm, 1.5 mm isotropic, 2 acquisitions along positive and negative phase-encoding directions).

#### MRI pre-processing

This will include non-uniformity correction (Sled and Pike, 1998), spatial standardization and brain extraction (excluding the skull). To ensure the brain structure volumes are comparable among subjects, the MRI data of each subject will be transformed from the original space to a common stereotactic space using multi-scale affine registration (Kannala et al., 2005). Brain regions will be automatically segmented from the head MRI data using the brain extraction tool (Smith, 2002).

#### Brain segmentation

Brain tissue will be classified into gray matter, white matter and cerebrospinal fluid (Cocosco et al., 2003). Whole-brain segmentation will be achieved using an atlas-based approach (Han and Fischl, 2007), which automatically adjusts the existing atlas intensity model to newly inputted data. The ROI and other brain regions (parietal lobe, occipital lobe and basal ganglia) will be segmented and their volumes quantified using the Talairach brain atlas (Lancaster et al., 2007) and Demon registration (Thirion, 1998).

#### Infarct segmentation and quantification

Infarcts will be delineated semi-automatically as high-intensity regions on DWI images and WMH as high-density regions on FLAIR images (and isointense on T1 weighted images) using ITK-SNAP software. The segmented infarct and WMH regions will be combined with the ROI and other brain-region masks generated in the previous step. The infarct and WMH pixels that fall within the ROI and other brain regions can then be calculated.

#### Diffusion MRI

The fiber orientation density function will be estimated from diffusion MRI data using FSL’s BEDPOSTX. A dense connectome containing the number of fiber tracks between a pair of greyordinates will be subsequently obtained using probabilistic tractography using FSL’s PROBTRACKX. The dense connectome will then be parcellated using the Harvard-Oxford cortical and subcortical atlas. Diffusion metrics, such as fractional anisotropy, mean diffusivity, diffusional kurtosis, etc., of each of the fiber tracts underlying this parcellated connectome will also be estimated.

### Statistical analysis

All of the variables will be tested for normality using Kolmogorov–Smirnov tests with a significance threshold of *p* < 0.05. We will first compare the IRI and other neuropsychological test scores between stroke subjects and normal controls. To examine the correlates of LoE, the demographic, clinical and MRI variables (age, gender, NIHSS, AES, GDS, BDI, MoCA, ROI infarcts, microbleeds and WMH volumes) will be compared between patients with and without LoE at T1 using the χ^2^ test, Student’s t test or Mann Whitney U test, as appropriate. A stepwise logistic regression will be performed to assess the importance of lesions in the ROIs, together with other significant variables in the above univariate analyses. In this regression, the independent variables are presence of lesions in ROIs and significant demographic, clinical and MRI variables, whereas the dependent variable is the presence of LoE. For patients with LoE at T1, the IRI total and subscale scores for the groups with/without ROI infarcts will be compared using t-test. In this analysis, the independent variables are the presence of ROI infarcts, dependent variables are the IRI scores.

To examine neuropsychological functions in LoE, the performance of the LoE group, non-LoE group and normal controls on emotional processing and ToM tasks and executive and basic cognitive function tasks will be compared using analysis of variance. The correlation between the IRI total and subscale scores and the performance of the LoE group on the above neuropsychological tests will be computed using Pearson’s or Spearman’s correlation coefficients, as appropriate. Finally, regression analysis of the IRI total and subscale scores will be performed using the significantly correlated neuropsychological functions as predictors. In the above analysis, the independent variables are the scores of emotional processing and ToM tasks and executive and basic cognitive function tasks, dependent variables are the IRI total and subscale scores.

To examine the predictors of LoE remission, the demographic, clinical and MRI variables of remitters and non-remitters at T2/T3 will be examined by logistic regression. We will also test a series of generalized estimating equation models to evaluate the association between clinical and brain MRI characteristics and risk of LoE across all follow-up assessments (T1/T2/T3). First, we will run a univariate model to fit a logistic regression. Next, we will examine the association between the demographic variables, concurrent medical conditions and risk of LoE. The second model will add baseline IRI scores, NIHSS scores and cognitive function scores. The brain MRI characteristics will be entered in the final model. The level of significance will be set at 0.05. In the above analysis, the independent variables are demographic variables, concurrent medical conditions, baseline IRI and EQ scores, NIHSS scores and cognitive function scores, and brain MRI characteristics, dependent variable is remission of LoE. The above analysis will be repeated for the Diffusion MRI metrics.

## Discussion

We try to achieve a homologous patient population by narrowing the age, ethnicity, handedness and duration of LoE. Patients with other causes of LoE, such as psychiatric or neurological disorders are excluded. This project will be the first longitudinal study to examine the role of VMPC, OFC, IFC, ACC, ATL, anterior insula, amygdala and thalamus in a large sample of consecutively admitted stroke survivors with LoE. The results will shed light on the association between the above brain regions and LoE risk. They are thus likely to be applicable to the large population of neurological patients at risk of LoE and should also stimulate further research in this field.
